# Analyzing the Carbon Footprint of an Intravitreal Injection

**DOI:** 10.18502/jovr.v16i3.9433

**Published:** 2021-07-29

**Authors:** Barry Power, Robert Brady, Paul Connell

**Affiliations:** ^1^Vitreoretinal Department, Mater Misericordiae University Hospital, Dublin, Ireland

**Keywords:** Anti-VEGF, Climate Change, Medical Retina, Sustainability

## Abstract

**Purpose:**

To estimate the carbon footprint of a single intravitreal injection in a hospital-based intravitreal service.

**Methods:**

Greenhouse gas emissions attributable to the delivery of an intravitreal injection were calculated using a hybrid lifecycle analysis technique. Data were collected regarding procurement of materials, patient travel, and building energy use.

**Results:**

Carbon emissions associated with a single intravitreal injection, excluding the anti-VEGF agent, were 13.68 kg CO ${}_{2}$ eq. This equates to 82,100 kg CO ${}_{2}$ eq annually for our service. Patient travel accounted for the majority of emissions at 77%, with procurement accounting 19% for and building energy usage for 4% of total emissions. The omission of items considered dispensable from injection packs would reduce carbon emissions by an estimated 0.56 kg per injection – an annual saving of 3,360 kg CO ${}_{2}$ eq for our service. Similar savings, if extrapolated to a country the size of the United Kingdom, could yield annual carbon savings of 450,000 kg CO ${}_{2}$ eq. For context, a single one-way economy transatlantic flight produces 480 kg CO ${}_{2}$ eq per person.

**Conclusion:**

Wasteful practice in healthcare increases greenhouse gas production and drives climate change. The healthcare sector should be a leader in sustainable practice promotion and changes to high volume procedures have the largest impact on emissions. Long-acting agents offer the greatest future potential for meaningful reductions.

##  INTRODUCTION

Climate change is a serious global threat and the healthcare industry is a large net contributor. The climate of the earth has changed throughout history but the changes over the last 50 years are unprecedented for millennia. The National Aeronautics and Space Administration (NASA) states that this change is highly likely ( > 95% probability) to be attributable to human activity and this is supported by multiple studies with the wider scientific community in agreement.^[1,2,3,4]^ This change is rapid with the current rate of warming 10 times faster than similar historical periods.^[5]^ Climate change will disproportionately affect developing countries.^[6]^

Greenhouse gases (GHGs) are those considered to be causative of global warming and carbon footprinting is the commonly accepted scientific methodology for quantifying the volume of GHGs a product or activity produces. Carbon footprints typically break a process down into components, which are felt to contribute significantly to GHG production. The footprint puts particular emphasis on the emissions that are felt to drive climate change. Four gases (carbon dioxide, methane, nitrous oxide, and Sulphur hexafluoride) and two groups of gases (hydrofluorocarbons and perfluorocarbons) are described in the Kyoto protocol as contributing to global warming. Carbon dioxide (CO ${}_{2}$ ) is used as the reference gas with the total emissions expressed in units called carbon dioxide equivalents (CO ${}_{2}$ eq).^[7]^

In the United Kingdom (UK) and the United States of America (USA), healthcare is responsible for 4% and 10% annual emissions respectively.^[8,9]^ Amongst the public sector in the UK, healthcare is estimated to account for 25% of total emissions.^[10]^ Limited studies to date have described the carbon footprint of various healthcare processes.^[11,12,13]^ In the ophthalmic literature, the carbon footprint of the cataract surgery pathway in a unit in the UK has been estimated to be 181 kg CO ${}_{2}$ eq.^[14]^

The UK National Health Service (NHS) aims to be a leader among global healthcare providers in facing up to its responsibilities concerning carbon emissions. The NHS carbon reduction strategy sets an ambition for the healthcare sector to drive the change toward a low carbon society.^[15]^ Taking 2007 levels as the baseline, the strategic goals are a 34% reduction in carbon production by 2020 and an 80% reduction by 2050.

Efforts to reduce healthcare-related carbon emissions are most effective when focused macroscopically on processes. High volume procedures, such as intravitreal injections, offer scope for meaningful net emission reductions with procedural changes.

With an ageing population, the number of intravitreal injections performed continues to grow and an estimated 5.9 million intravitreal injections were performed in the USA in 2016.^[16]^ In the UK, injection procedures increased by 215% between January 2010 and May 2014.^[17]^

Our aims for this paper are to:

• estimate the overall carbon emission per injection;
• understand where in the process the carbon is emitted;
• identified hotspots within this process can then be targeted to achieve the most effective reductions.

Our target audience is those working in the healthcare sector including managers and clinicians.

##  METHODS

There are two main methods of carbon footprinting – lifecycle analysis (LCA) and input–output-based analysis. A hybrid LCA utilizes both methods in an attempt to address the inherent limitations of the two systems. We discuss our rationale for using this technique at the end of the section. The study was performed in the Mater Misericordiae University Hospital Dublin from June to August in 2018. This study adhered to the guidelines of the Declaration of Helsinki. All methods of carbon emission estimation must have a scope or boundary. For this study, we calculated emissions from three main components of the intravitreal injection process: (1) procurement of the materials utilized; (2) patient travel to and from the hospital; and (3) building energy use.

Greenhouse gas (GHG) emissions were converted to kilograms carbon dioxide equivalent (kg CO ${}_{2}$ eq) which is the accepted common unit for measuring GHG production. This measure is the amount of carbon dioxide that would have the same global warming potential as the GHGs produced by the process measured.

### Procurement 

Procurement emissions are defined as the emissions associated with all of the elements of production, distribution, consumption, and disposal of an item.

#### Production, Distribution, and Consumption of Materials 

Carbon emissions associated with production, distribution, and consumption were calculated using an input–output model. The input–output method is used when a lifecycle-based assessment is impractical due to difficulty in assigning boundaries to complex processes with many direct and indirect components. This form of carbon estimation uses the monetary cost of a product or process to estimate carbon emissions. Different conversion factors exist for different industries; a conversion factor specific to the medical industry was used for the injection pack and a pharmaceutical conversion factor for comparison of the different agents. These carbon emission conversion factors were obtained from the 2011 UK Department of Environment and Rural Affairs (DEFRA) emission modelling documents, using the 2018 price estimates.^[19]^ The cost of the injection pack utilized was €10 ( £ 9.10). The local costs of Bevacizumab, Ranibizumab, and Aflibercept were €44, €820, and €1100, respectively – each purchased in prefilled syringes. These syringes are single dose units and come separately from the injection packs.

#### Waste Disposal

The carbon emissions associated with waste disposal were calculated using a lifecycle-based analysis. This type of analysis collects all of the materials utilized in a process and estimates emissions attributable to each item. The calculations were performed using the 2018 UK government GHG conversion publication.^[20]^ The calculation is weight-based. Each separate product used for delivering an intravitreal injection was weighed and its composition classified according to the document. Conversion factors were used to convert this data into kg CO ${}_{2}$ eq. The conversion factors consider the composition of the product disposed of and the method of disposal (clinical incineration or landfill). The conversion factor for incineration of clinical/hazardous waste (which is undertaken at higher heat and without energy extraction) was taken to be 1,833 in accordance with previous studies.^[14]^ Each healthcare provider performing injections in the service was surveyed (*n* = 6) and asked to document which waste disposal bin each item was placed in after use. The method of disposal was taken to be the majority result. The contents of the pack are summarized in Table 3 in the Results section.

### Travel

Emission production attributable to patient travel to and from the hospital was calculated based on the distance travelled (hospital to home address) and the mode of transport. Patients were consented for participation. Google maps distance was used to calculate the distance travelled by the patient (www.googlemaps.ie). One hundred sequential patients were surveyed to document mode of travel and journeys were taken to be round-trips. The 2018 UK government GHG conversion publication was used to convert this data into kg CO ${}_{2}$ eq per km travelled.^[20]^

### Energy Usage

Data pertaining to energy usage in the study hospital was not available so Moorfield's Eye Hospital was used as a representative substitute. The building energy use was estimated in a similar manner to previous studies.^[14]^ Energy use per m ${}^{2}$ of floor space was estimated using the UK 2017 National Estates Return Information Collection database.^[21]^ This data was then converted to kg CO ${}_{2}$ eq per kW using the carbon conversion factor. The 100m ${}^{2}$ floor space utilized by the intravitreal injection service was defined as the check-in/waiting room and a two-bed laminar airflow injection suite. Energy usage was taken to be that of standard clinical floor space. The waiting room is used for a variety of different purposes, so its contribution was adjusted accordingly. Energy use per injection was calculated by estimating the proportional energy use of the floor space when utilized by the injection service and dividing it by the total number of injections performed per annum (6,000). The injection suite is not used for other purposes. This data is summarized in Table 1.

**Table 1:** Emissions factors


**Procurement**	**Emission Factor (kg CO ${}_{2}$ eq per £ )**
**Pharmaceutical**	0.43
**Medical**	0.28
**Waste Disposal **	**Emission Factor Range (kg CO ${}_{2}$ eq per ton)**
**Landfill**	9-445
**Incineration**	1833
**Travel Method**	**Emission Factor (kg CO ${}_{2}$ eq per Km)**
**Car**	0.178
**Bus**	0.101
**Train**	0.044
**Walk/Cycle**	0
**Energy Use**	**Emission Factor (kg CO ${}_{2}$ eq per KWh)**
**Electricity**	0.59
Kg CO2eq, kilograms of carbon dioxide equivalents; Km, kilometer; KWh, kilowatt hour	

**Table 2:** Patient travel emissions


	**Car**	**Bus**	**Train**	**Walk/cycle**
**Conversion Factor**	0.1778	0.11	0.044	0
**Average Distance (km)**	33	51	42	NA
**Patients**	64	29	3	4
Km, kilometers				

**Table 3:** Intravitreal injection pack contents and waste disposal emission data


**Item**	**Weight (g)**	**Classification**	**Landfill**	**Incineration**	**Conversion Fx**	**kg CO ${}_{2}$ eq**
NaCl	14.68	Plastic rigid	6	0	9	0.0001
*Paper Towel*	*20.84*	*Cloth*	*6*	*0*	*445*	*0.009*
Sterile Gloves	31.58	Plastic film	6	0	9	0.00003
Drape	19.79	Plastic film	0	0	9	0.0002
Scleral Marker	1.76	Plastic rigid	2	4	1833	0.00004
Speculum	2.8	Metal	1	5	1833	0.00006
Wooden Cotton Buds x 5	4.08	Wood	2	4	1833	0.00009
*2 ml syringe*	*2.72*	*Plastic rigid*	*6*	*0*	*0*	*0.00002*
Syringe	3.71	Plastic rigid	0	6	1833	0.00008
Bevacizumab Needle	0.59	Metal	0	6	1833	0.00001
Gauze Square x 4	7.77	Cloth	6	0	445	0.004
*Plastic Forceps*	*9.64*	*Plastic rigid*	*2*	*4*	*1833*	*0.0002*
Iodine minim	1.5	Plastic rigid	6	0	9	0.00001
Plastic container 30 ml	3.31	Plastic rigid	6	0	9	0.00003
Plastic container 30 ml	3.31	Plastic rigid	6	0	9	0.00003
G Proxymetacaine	1.59	Plastic rigid	6	0	9	0.00001
G Chloromycetin	1.59	Plastic rigid	6	0	9	0.00001
*Plastic Container Outer*	*27.85*	*Plastic rigid*	*6*	*0*	*9*	*0.00003*
Outer Wrapping	37.5	Plastic film	6	0	9	0.0003
Fx, conversion Factor; Gs, weight in grams conversion; Kg CO2eq, kilograms of carbon dioxide equivalents						

Clinicians perform intravitreal injections with a surgical scrub to the elbow before an injection list. One facemask is used for a list. Attire is typically office attire with one plastic apron per list. Hands are sterilized between cases with a betadine scrub or alcohol hand gel. All clinicians use separate sterile gloves and an individual intraocular injection pack for each patient. The contents of the injection pack and their associated data are summarized in the Results section in Table 3. Only items used for each separate injection were considered for analysis (facemask and apron used for the whole list, therefore not included).

#### Inclusions

Travel of patients to and from the hospital was included in the study. This maintains consistency with previous studies.^[11,12,13][14]^ Although it does not follow the PAS 2050 guidelines, we believe that inclusion transport in this hybrid LCA is appropriate. Patient travel is essential to the procedure and represents a large component of the carbon production. Due to the frequent and recurrent nature of intravitreal injections, travel is an important source of emissions.

#### Exclusions 

Excluded components include but are not limited to: items deemed negligible to overall emissions score (e.g., ink, medical record documents), procurement of items used over the medium to long-term (computers, injection beds, diagnostic tools, e.g., OCT, etc.), human inputs into the system, building/construction costs, food and drink, staff training, and research.

#### Methodology Discussion

We utilized a hybrid LCA for the calculation of this carbon footprint. The main advantage of this methodology is that it provides a more comprehensive analysis than other techniques.^[22,23]^ It helps to reduce the impact of truncation error, which can be caused by the boundary placement required in PAS 2050 adhering LCAs. The disadvantage with this methodology is it can be less reproducible – particularly when the methodology is poorly defined. This can be problematic in formal industry assessments as it can make a direct comparison between processes more difficult. This study aimed to make an all-encompassing, accurate estimate of the component and total carbon production; for this, we felt the hybrid methodology was the most robust.

Regarding the use of input–output models to calculate carbon emissions, values are determined by the cost of materials. Naturally, high cost materials will have large carbon estimates. These values can seemingly overestimate emissions. However, pharmaceutical agents, for example, require extensive research and development before coming to market. Marketing of these agents, drug representative activities, and education of clinicians and patients are key components of the industry. All of these activities create emissions. Thus, just as the monetary cost of these critical components of drug development are embedded in the cost of the product, it can be argued that the downstream carbon emissions are also embedded within the cost of the product. We produced estimates excluding and including the pharmaceutical agents due to their very large contribution to emission volumes. We did not consider potential/debatable differences in duration of action between agents to be a significant factor in emissions production.

##  RESULTS

**Figure 1:**
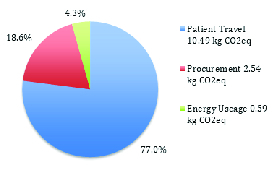
Proportional carbon emissions. Kg CO2eq, kilograms of carbon dioxide equivalents

One intravitreal injection, excluding the anti-VEGF agent, was calculated to produce 13.68 kg CO ${}_{2}$ eq. The breakdown for the different contributions from the three main sources is outlined in Figure 1.

**Figure 2:**
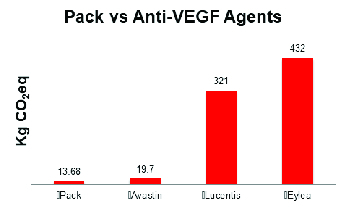
Estimated carbon emissions associated with the injection pack, Avastin, Lucentis, and Eylea. Kg CO2eq, kilograms of carbon dioxide equivalents

The single largest contributor to the carbon footprint was patient travel at 10.49 kg CO ${}_{2}$ eq (77%) per injection. The majority of patients travelled to and from their appointments by car. Our service is situated in a tertiary referral center and our catchment area is the northern half of the province of Leinster. The average one-way distance travelled was 38 km. Table 2 outlines the travel emission conversion factors and the breakdown of the common modes of transport to the hospital.

Medical procurement (the injection pack) accounted for 2.54 kg CO ${}_{2}$ eq (19%). Despite a large amount of physical waste generated per intravitreal injection, the carbon emissions produced through waste disposal were negligible – just 0.05 kg CO ${}_{2}$ eq per injection or 0.04% of the total emissions.

The contents of the injection packs, the method of waste disposal and the emissions data are summarized in Table 3. Materials deemed to be surplus to requirements (by > 75% of clinicians) are highlighted in *bold* *italics*.

When the different anti-VEGF agents are included, emission estimates vary widely. Using input–output analysis, bevacizumab, ranibizumab, and aflibercept procurement produce an estimated 20, 320, and 423 kg CO ${}_{2}$ eq, respectively, per injection. Figure 2 compares the emissions estimates of the anti-VEGF agents to those of the pack.

### Opportunities for Carbon Reductions

No immediate or realistic opportunity for carbon savings related to building energy use was identified. If the service facilities/logistics allow, patients can receive injections on the same day as clinic appointments, reducing overall hospital attendances and lowering emissions. The use of bilateral same-day injections is another potential mitigation strategy. Bilateral same-day injections are safe, however, patient safety and medico-legal concerns persist.^[24,25]^

We identified the procurement of the injection pack as an additional opportunity to reduce emissions. A survey of the clinicians performing the injections identified several items that were deemed to be safely dispensable from the intravitreal packs – these were: plastic tongs for betadine application, a 2 ml syringe, the hard outer plastic container, and the paper hand towel. Betadine is generally applied to the patient's skin using a cotton gauze held by hand, the 2 ml syringe was not used and the hand towel was not necessary because alcohol was used rather than hand washing to sterilize the hands between patients. Removal of these items from our packs achieved a price reduction of €2.05 per pack, negotiated with our supplier. Using the input–output model for production, distribution, and consumption and the lifecycle-based analysis for disposal, this equates to 0.56 kg CO ${}_{2}$ eq per injection (4%) or an overall reduction of 3,360 kg CO ${}_{2}$ eq per annum for our hospital-based intravitreal service (based on our annual provision of 6,000 injections).

##  DISCUSSION

Discussion around the importance of sustainability in ophthalmology has increased over the past decade.^[26,27]^ To our knowledge, this is the first study to estimate the carbon emissions attributable to intravitreal injections. This intervention has rapidly become the most common invasive procedure performed in ophthalmology.^[28]^ An intravitreal injection is a typical medical procedure. We hope that our breakdown of the sources of emissions, from the different components of the procedure, will help to identify the most suitable targets for carbon reduction strategies.

Current estimations of international rates of intravitreal injections differ. Data from the USA estimates the 2013 rate at 130 injections per 100,000 population.^[16]^ The USA estimates project a 10–20% per annum increase in numbers of injections performed. If we use an estimate of 15% and adjust the UK 2015 estimate of 714 per 100,000, the estimated rate for 2018 is 1,248 per 100,000. For the current UK population (66 million), this amounts to 824,000 injections. Though pack contents will differ across units, similar changes to contents of injection packs used in the UK could yield a substantial reduction (approximately 460 ton CO ${}_{2}$ eq). To put the figures into context, a one-way, economy class transatlantic flight from London to New York City produces an estimated 480 kg CO ${}_{2}$ eq per person.^[29]^

In addition to the reductions possible in carbon emissions, the judicious omission of unnecessary single-use plastics from routine procedure packs has other environmental benefits. The European Union (EU) has recently followed some countries in banning the use of several forms of single-use plastics.^[30]^ Plastic does not biodegrade and there are an estimated 269,000 tons of plastic floating in the ocean.^[31]^ Three of the four dispensable items in our packs are hard plastic. Dialogue with manufacturing companies may be able to further reduce the plastic contents of the packs through omission of unnecessary items. The annual weight of the dispensable plastic in the packs used by our service is over 240 kg.

In 2013, Morris et al estimated the carbon emissions attributable to the Cardiff cataract pathway to be 181 kg CO ${}_{2}$ eq, with an annual production of 63 mega ton kg CO ${}_{2}$ eq in England.^[14]^ The proportional breakdown of the components of the cataract pathway differs slightly from that of an intravitreal injection. Procurement was the largest proportional contributor in the cataract pathway at 53.8%. Procurement would also be the highest proportional contributor in the intravitreal injection footprint should the anti-VEGF agents use be included. Proportional energy use was understandably greater for the cataract surgery pathway (36.1% vs 4%), as was the contribution of waste disposal (1.6% vs 0.05%). Proportional travel estimates were less in the cataract surgery pathway (10.1% vs 76.6%) but gross travel emissions were greater (18.3 vs 10.49 kg CO ${}_{2}$ eq). The cataract pathway included two separate round-trip journeys per patient, potentially explaining the higher gross figure.

Our study has allowed us to identify some easily implemented changes, which would achieve a significant carbon reduction if extrapolated to a national level and is an example of a “bottom-up” change that is immediately realizable. The far larger contribution of the production, distribution, and consumption elements of procurement, compared with the waste disposal element, highlights the need to target emission generation at source. Efforts to recycle (challenging in healthcare due to infection control concerns) and improve waste management are likely to be small in comparison. The omission of unnecessary items eliminates all of the emissions embedded in the procurement process of an item.

Our study has some limitations. As demonstrated by the comparison of cataract surgery and intravitreal injection, carbon estimation is, by its nature, variable. It is impossible to measure every direct and indirect contribution to these complex processes. Our boundaries exclude known and unknown sources of carbon emissions. Methodologies for carbon estimation are not designed with specific industries in mind and interpretations of models can differ. Patient travel estimates reflect the local geography and the boundaries of the studied catchment area; thus, variation is expected. Calculations relating to the production of materials (or the “procurement” component of this footprinting analysis) may under and overestimate emissions as the estimates are based on price alone. These estimates are based on production in broadly defined industries, not directly on ophthalmology. We recognize the limitations of our estimates based on input–output data but feel that they are sufficiently accurate to satisfy our aims.

Other studies have estimated the GHG production associated with the production of pharmaceutical agents. Parvatker et al recently performed a component LCA of several inhaled anesthetic agents using a chemical-engineering scale-up technique.^[32]^ This is a more accurate measure of the emissions associated with the simple production of a volume of a drug. It does not, however, consider the background emissions associated with research and development, marketing, etc. In addition, it is a technique, which is very complex to perform so it may be difficult for clinicians and non-specialists to replicate it.

If sustainability is a goal of the wider ophthalmology community, intravitreal injections, due to their exponential increases in volume, should be a prime target for such efforts. Despite some limitations, we feel our estimation is a good guide for the component breakdown of the carbon emission profile of an intravitreal injection. The calculation of a carbon footprint is clearly not necessary for every individual procedure. The results not always intuitive and analysis of a process can give us valuable insight into where to focus on reduction strategies. We echo Morris et al in stressing the importance of focusing on procurement while again demonstrating the somewhat limited potential impact of waste recycling strategies.^[14]^ In the future, the introduction of long-acting agents may reduce the number of injections performed.^[33]^ This represents the greatest potential to achieve large scale emissions reductions associated with intravitreal injections as it would tackle both procurement and travel associated emissions. Extensive carbon savings would be achievable, not to mention the obvious economic and service provision benefits.

For many clinicians, the large quantity of GHG produced by our health sector may seem outside of their direct control or responsibility. Nonetheless, clinicians should recognize that they can make simple changes that can have an immediate positive impact on carbon emissions. We must lead by example and address the harmful effects of mindless wastefulness in our daily practice. Sensible alterations to pack contents can have economic benefits, as well as lowering GHG production. These reductions can have a large overall impact when extrapolated to a service or even a national level. In addition to lowering GHG production, the reduction in single-use plastics has other environmental benefits beyond the scope of this study. Pre-prepared packs are in widespread use for many minor procedures across healthcare, and our study may encourage other clinicians to make similar choices. We strongly advocate bottom-up interventions to drive an overall reduction in carbon emissions in ophthalmology and healthcare as a whole.

##  Financial Support and Sponsorship

None.

##  Conflicts of Interest

The authors declare no conflicts of interest.
